# From Conventional to Next-Generation Strategies: Recent Advances in Polymeric Micelle Preparation for Drug Delivery

**DOI:** 10.3390/pharmaceutics17101360

**Published:** 2025-10-21

**Authors:** Suhyeon Cho, Morteza Rasoulianboroujeni, Rae Hyung Kang, Glen S. Kwon

**Affiliations:** 1 Department of Pharmaceutical Engineering, Dankook University, Cheonan 31116, Republic of Korea; julie3474@naver.com; 2 Pharmaceutical Sciences Division, School of Pharmacy, University of Wisconsin, 777 Highland Avenue, Madison, WI 53705, USA; 3 Department of Pharmaceutical Sciences, Gatton College of Pharmacy, East Tennessee State University, Johnson City, TN 37614, USA; rasoulianbor@etsu.edu

**Keywords:** polymeric micelles, micelle fabrication methods, drug delivery systems, scalable nanomanufacturing, stimuli-responsive micelle

## Abstract

Polymeric micelles are promising nanocarriers for hydrophobic drug delivery, offering enhanced solubility, circulation time, and targeted release. This review presents a comprehensive evaluation of micelle preparation strategies, spanning conventional methods such as direct dissolution, dialysis, and thin-film hydration to emerging techniques including microfluidics, supercritical fluids, stimuli-responsive systems, and PEG-assisted assembly. Each method is compared in terms of scalability, reproducibility, solvent use, and regulatory compatibility. Among them, PEG-assisted methods show particular promise due to their simplicity and industrial readiness. We also explore the impact of fabrication strategy on drug loading, stability, and therapeutic efficacy across applications in cancer, infection, and inflammation. Finally, the review discusses key challenges in storage, manufacturing, and regulation, and highlights potential solutions through Quality-by-Design and scalable process integration. These insights provide guidance for the rational development of clinically translatable micelle-based drug delivery systems.

## 1. Introduction

Nanoparticle-based drug delivery systems (DDS) have revolutionized the landscape of pharmaceutical development by offering enhanced therapeutic precision, improved pharmacokinetics, and the potential for targeted delivery [1,2,3,4,5,6,7,8]. These nanoscale platforms ranging from liposomes and dendrimers to polymeric nanoparticles and micelles enable the encapsulation and transport of bioactive compounds with challenging physicochemical properties [1,2,9,10,11,12]. Among them, polymeric micelles have garnered particular attention for their capacity to solubilize poorly water-soluble drugs while maintaining high colloidal stability and biocompatibility [7,13,14,15].

Within this broader context, and alongside liposomes, dendrimers, and nanogels, polymeric micelles offer a distinct combination of low critical micelle concentration, efficient solubilization of poorly water soluble drugs, and modular polymer design [16,17,18]. Compared with vesicular liposomes and crosslinked gels, micelles can be formulated with streamlined workflows and readily tunable chemistries, aligning with this review’s focus on manufacturing and translation [19,20]. In this review we focus on method-level choices that enable manufacturing and translation from conventional methods to emerging technique such as microfluidic assembly, supercritical fluid processing, and PEG-assisted routes, which reduce solvent burden, support lyophilization, and can be implemented with quality-by-design (QbD) and process analytical technologies (PAT) (Section 3.6 and Section 4.5).

Polymeric micelles, as self-assembled colloidal systems formed from amphiphilic copolymers (most commonly block copolymers) in aqueous environments, possess a distinctive core–shell architecture [9,10,21,22]. This structure facilitates the encapsulation of hydrophobic therapeutics within the core while maintaining a hydrophilic corona that imparts circulation stability and stealth characteristics [10,13,21]. Their physicochemical tunability, passive targeting capabilities through the enhanced permeability and retention (EPR) effect, and modular design potential have positioned them as versatile platforms within the field of nanomedicine [10,13,21,23].

Numerous studies have demonstrated that polymeric micelles can significantly enhance drug pharmacokinetics, reduce off-target toxicity, and improve therapeutic efficacy [9,10,13,21,24]. Several micellar formulations have advanced to clinical trials or achieved commercial approval, highlighting their translational potential [25,26]. Nonetheless, broader clinical adoption remains challenged by issues related to manufacturing reproducibility, scalability, and regulatory acceptance. Many current micelle systems depend on traditional fabrication techniques such as solvent evaporation, thin-film hydration, dialysis, and co-solvent exchange, which often involve organic solvents, multi-step procedures, and batch-dependent variables [27,28,29,30,31,32,33]. These limitations can adversely affect micelle uniformity, drug loading efficiency, stability, and overall cost-effectiveness, thereby hindering their practical deployment.

To address these shortcomings, recent research has focused on developing novel micelle preparation methods aimed at enhancing scalability, reducing or eliminating organic solvent use, streamlining manufacturing workflows, and improving formulation robustness [34,35,36,37]. Emerging strategies including continuous flow processing, stimuli-responsive self-assembly systems, and hybrid material integrations have significantly broadened the micelle design landscape and facilitated their application in advanced drug delivery contexts [38,39,40,41]. These include transdermal, oral, and inhalable routes, as well as multifunctional platforms with environmental responsiveness and combinatorial therapeutic functions [42,43,44,45,46,47,48,49,50,51,52,53,54].

Although numerous reviews have explored the biomedical applications and polymer chemistries of polymeric micelles, a critical and systematic evaluation of micelle preparation methodologies, especially in terms of scalability, formulation consistency, and pharmaceutical translation, remains lacking. Existing literature often marginalizes fabrication techniques or addresses them within narrow technological silos, without fully contextualizing their development in relation to evolving clinical and industrial demands. Notably, prior surveys have rarely integrated regulatory and pharmaceutical-quality considerations into method-centric comparisons (e.g., residual-solvent limits, batch-to-batch variability under QbD/PAT frameworks, and readiness for continuous manufacturing) leaving the translational consequences of fabrication choices underexplored.

**Figure 1:**
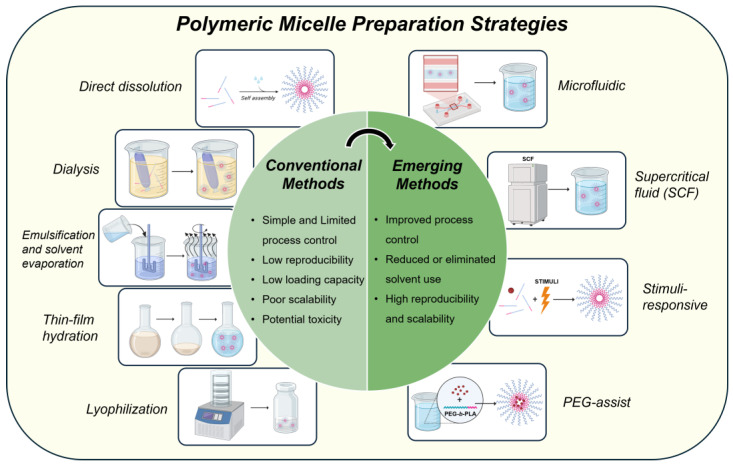
Overview of polymeric micelle preparation strategies. Polymeric micelles can be prepared via various strategies categorized into conventional and emerging methods. Conventional approaches such as direct dissolution, dialysis, emulsification and solvent evaporation, thin-film hydration, and lyophilization offer simplicity but often suffer from limited process control, low reproducibility, poor scalability, and potential toxicity due to the use of organic solvents. In contrast, emerging methods including microfluidic mixing, supercritical fluid (SCF) processing, stimuli-responsive systems, and PEG-assisted techniques offer improved control over physicochemical parameters, reduced or eliminated use of harmful solvents, and enhanced scalability and reproducibility. These advancements contribute to the development of micellar systems more suitable for clinical translation and pharmaceutical manufacturing.

This review aims to fill that gap by presenting a comprehensive, methodologically focused analysis of polymeric micelle preparation strategies (Figure 1). By chronologically organizing these methods from conventional to next-generation approaches, we seek to elucidate how advances in fabrication directly influence key attributes such as drug loading capacity, particle size uniformity, structural stability, and clinical applicability. Particular emphasis is placed on emergent methods with demonstrated potential for scalable, reproducible, and pharmaceutically compliant production, as these are poised to define the next generation of clinically relevant micellar drug delivery systems.

Throughout, we evaluate preparation methods using manufacturing indicators such as size and PDI variability, drug-loading reproducibility, residual-solvent control, process throughput, and readiness for continuous processing and PAT to foreground industrial scalability and quality. While block copolymers serve as the primary anchor for this review, we also consider graft copolymers where appropriate, because graft density and side chain length provide additional control over CMC, core packing, drug loading and release, and stability under dilution-factors directly tied to reproducibility and scale up (see Section 2) [55,56]. Through this lens, the review not only synthesizes the current landscape of micelle fabrication technologies but also critically evaluates their respective roles in enabling the future integration of micellar platforms into real-world therapeutic applications.

## 2. Fundamentals of Polymeric Micelles

**Figure 2:**
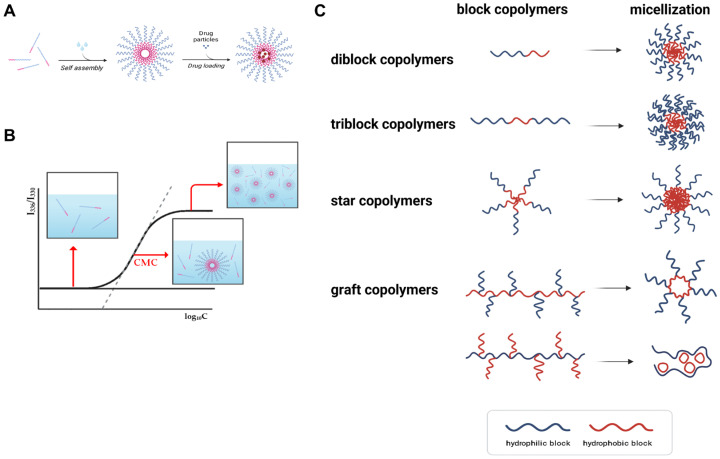
Fundamental principles of polymeric micelle formation and structural diversity. (**A**) Schematic illustration of polymeric micelle formation via the spontaneous self-assembly of amphiphilic block copolymers in aqueous solution. (**B**) As polymer concentration increases and surpasses the critical micelle concentration (CMC), hydrophobic interactions drive the formation of core–shell structures capable of loading hydrophobic drug molecules. The graph depicts the relationship between light scattering intensity and logarithmic polymer concentration, indicating micelle formation above the CMC threshold. (**C**) Representative architectures of block copolymers used in micelle formation. These include diblock copolymers, triblock copolymers, star copolymers and graft copolymers. Right side represents micelles in which each block copolymer is formed through self-assembly in aqueous solution. Polymer architecture plays a key role in determining micelle size, stability, and drug loading behavior.

Polymeric micelles are nanoscale supramolecular assemblies formed via the spontaneous self-association of amphiphilic block copolymers in aqueous environments (Figure 2A) [9,10,14]. These structures emerge when the polymer concentration surpasses a critical threshold, known as the critical micelle concentration (CMC), beyond which unfavorable interactions between hydrophobic polymer segments and water drive the thermodynamically favorable formation of a core–shell architecture (Figure 2B). In this configuration, a hydrophobic core sequesters poorly water-soluble drugs, while a hydrophilic corona confers colloidal stability and mitigates recognition and clearance by the mononuclear phagocyte system [9,10,14].

The physicochemical characteristics and functional performance of polymeric micelles are highly dependent on the molecular design of their constituent copolymers (most commonly block copolymers) (Figure 2C). Hydrophilic segments are commonly derived from polymers such as poly(ethylene glycol) (PEG), poly(N-vinylpyrrolidone), or poly(2-oxazoline), while hydrophobic segments typically consist of biodegradable polyesters such as poly(lactic acid) (PLA), poly(ε-caprolactone) (PCL), or poly(amino acids) [9,10,13,14,23,24]. Key design parameters including the molecular weight ratio of hydrophilic to hydrophobic blocks, total chain length, and polymer architecture (e.g., linear, triblock, graft, or star) profoundly influence micelle size, drug loading capacity, and colloidal stability. In this context we use ‘star’ to denote multi-arm polymers from multifunctional initiators and ‘graft’ to denote side-chain architectures on a linear backbone. These architectures influence CMC and core packing parameters tightly coupled to process robustness and scale-up [55,57]. Furthermore, the interfacial compatibility between the drug and the hydrophobic block, as well as the rigidity of the core-forming segment, governs drug encapsulation efficiency and retention.

Typically, polymeric micelles possess diameters ranging from 10 to 100 nanometers, a size range conducive to exploiting the enhanced permeability and retention (EPR) effect for passive accumulation in tumor tissues [9,10,13]. Drug association with polymeric micelles can be achieved via three approaches: (i) physical encapsulation, (ii) chemical encapsulation by crosslinking the micellar core or shell, and (iii) covalent conjugation to the polymer [14,58,59,60,61]. Physical encapsulation relies on hydrophobic partitioning into the core, aided by π–π stacking and hydrogen bonding when applicable. It is operationally straightforward and broadly applicable but can be susceptible to drug leakage under dilution or protein mediated destabilization in systemic circulation [60]. While this method is operationally straightforward and broadly applicable, it may be susceptible to drug leakage under dilution or systemic circulation. Chemical encapsulation strengthens the micelle by crosslinking the core or the shell after or during self-assembly, thereby reducing dilution induced disassembly, lowering burst release, and improving redispersion and storage stability [59,60]. Crosslinking can be cleavable to permit on demand release or noncleavable for sustained retention; however, it introduces additional reagents and purification steps and may constrain process throughput unless optimized. In contrast, covalent conjugation involves the covalent attachment of drugs to the polymer backbone through cleavable linkers, allowing for more controlled and sustained release profiles [61]. Such systems are particularly advantageous in stimuli-responsive applications, where release is triggered by specific physiological cues such as pH shifts or redox gradients [36,40,62,63,64,65].

The in vivo stability and performance of polymeric micelles are determined not only by their static structural features but also by their dynamic behavior under physiological conditions [66]. A low CMC is generally preferred, as it promotes micelle integrity upon dilution in systemic circulation [67]. The hydrophilic corona acts as a steric barrier that reduces protein adsorption and macrophage uptake, thereby enhancing systemic circulation time and decreasing immunogenicity. Additionally, the degradation kinetics of the hydrophobic core influence drug release rates and the biocompatibility of degradation byproducts.

Recent advances in polymer chemistry have facilitated the development of multifunctional micelles that integrate targeting ligands, imaging agents, and stimuli-responsive moieties. These enhancements support site-specific delivery, non-invasive imaging, and controlled drug release in response to environmental triggers such as pH variation, enzymatic activity, or oxidative stress. Together, a fundamental understanding of micelle formation, structural dynamics, and drug loading mechanisms serves as a critical foundation for the rational design of next-generation micellar drug delivery systems.

## 3. Conventional Methods of Polymeric Micelle Preparation

**Figure 3:**
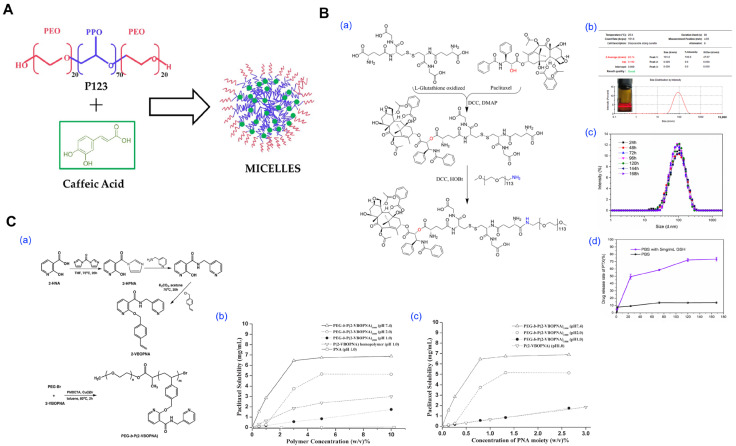
Representative experimental studies employing the direct dissolution method for polymeric micelle fabrication. (**A**) Self-assembly of Pluronic P123-based micelles loaded with caffeic acid (CA) for antioxidant stabilization. Schematic illustration of the formation of CA-encapsulated micelles via spontaneous aggregation of the amphiphilic triblock copolymer P123. Reproduced with permission from [28]. Copyright 2024 Multidisciplinary Digital Publishing Institute (MDPI). (**B**) Formulation and characterization of glutathione-responsive paclitaxel prodrug micelle based on GSSG. (**a**) Synthesis route of PTX-GSSG-PEG via direct dissolution. (**b**) Size distribution and polydispersity index (PDI) of PTX-GSSG-PEG. (**c**) The stability of particle size over time (24–168 h). (**d**) Drug release profile of PTX from PTX-GSSG-PEG micelles under PBS and PBS with 5 mg/mL glutathione conditions. Reproduced with permission from [75]. Copyright 2024 Multidisciplinary Digital Publishing Institute (MDPI). (**C**) Direct synthesis of diblock copolymers of PEG and P(2-VBOPNA) for aqueous solubilization of PTX. (**a**) Synthesis route of 2-(4-vinylbezyloxy)-N-picolylnicotinamide). (**b**) Evaluation of paclitaxel solubility at different pH conditions. (**c**) Evaluation of paclitaxel solubility at different pH conditions. Reproduced with permission from [74]. Copyright 2008 Elsevier.

**Figure 4:**
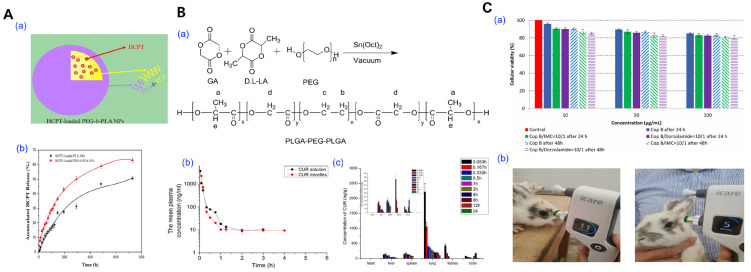
Representative examples of polymeric micelle fabrication via the dialysis method. (**A**) Comparative study of HCPT-loaded micelles prepared from PEG-*b*-PLA block copolymers and PLA homopolymers. (**a**) Schematic illustration of micellar self-assembly and drug entrapment during dialysis using PEG-*b*-PLA. (**b**) In vitro release profiles of HCPT in PBS (pH 7.4). Reproduced with permission from [76]. Copyright 2014 Springer. (**B**) Curcumin (CUR)-loaded PLGA-PEG-PLGA micelles fabricated via dialysis. (**a**) Synthetic route of the triblock copolymer PLGA-PEG-PLGA. (**b**) Plasma concentration–time curve following intravenous administration of free CUR or CUR-loaded micelles. (**c**) Biodistribution analysis across multiple organs of CUR-loaded micelles. Reproduced with permission from [77]. Copyright 2010 Elsevier. (**C**) Ocular delivery of dexamethasone via micelles formulated using dialysis. (**a**) Cell viability assays of micelles at varying concentrations and timepoints. (**b**) In vivo intraocular pressure (IOP) monitoring in rabbits. Reproduced with permission from [78]. Copyright 2022 Multidisciplinary Digital Publishing Institute (MDPI).

**Figure 5:**
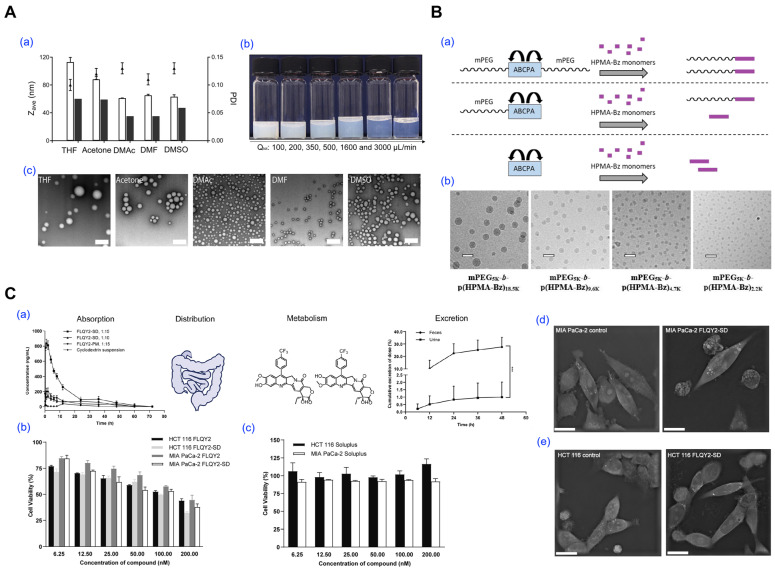
Representative studies utilizing emulsification and solvent evaporation methods for polymeric micelle preparation. (**A**) Impact of solvent selection and mixing parameters on micelle formation using solvent evaporation. (**a**) Particle size and polydispersity index (PDI) of p(HPMAm)_7.1k_-*b*-p(HPMAm-Bz)_15.0k_ micelles prepared with different organic solvents, as measured by DLS (open bars, *n* = 3) and TEM (filled bars, *n* = 1). (**b**) Macroscopic appearance of micelle dispersions produced at various mixing flow rates. (**c**) TEM images of micelles formed using THF, acetone, DMAc, DMF, and DMSO as polymer solvents (scale bar = 200 nm). Reproduced with permission from [31]. Copyright 2022 Elsevier. (**B**) Synthesis and morphological analysis of block copolymer micelles formed via solvent removal. (**a**) Polymerization routes yielding mPEG-*b*-p(HPMA-Bz) using different ABCPA-based initiators. (**b**) Cryo-TEM images of micelles with different PEG block lengths (scale bar = 50 nm). Reproduced with permission from [82]. Copyright 2018 American Chemical Society. (**C**) Emulsion-based formulation of FLQY2-loaded micelles and in vivo evaluation. (**a**) ADME (Absorption, Distribution, Metabolism, Excretion) evaluation of Soluplus^®^ micelles loaded with FLQY2. (*** *p*  <  0.001) (**b**) In vitro cytotoxicity of free FLQY2 versus micelle formulations in HCT116 and MIA PaCa-2 cell lines. (**c**) In vitro cytotoxicity comparison of Soluplus micelles in HCT116 and MIA PaCa-2 cells. (**d**) SEM images of MIA PaCa-2 and (**e**) HCT116 cells upon treatment, comparing with control and FLQY2-loaded micelles. Scale bars are 20 μm. Reproduced with permission from [83]. Copyright 2022 BioMed Central (BMC).

**Figure 6:**
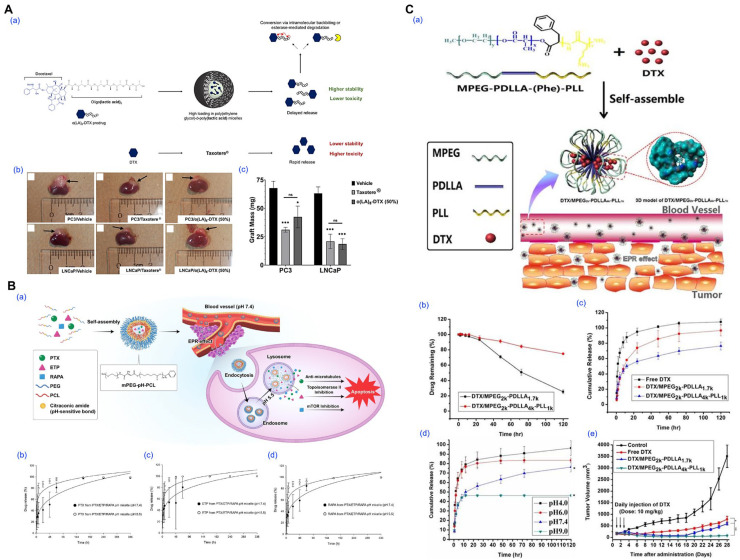
Representative examples of polymeric micelles prepared via thin-film hydration method and their biological performance. (**A**) Prodrug-based stabilization and delivery of docetaxel (DTX) using PEG-b-PLA micelles. (**a**) Schematic illustration of the structural features and hydrolytic activation of o(LA)_8_-DTX compared to free DTX (Taxotere^®^). Conjugation of the o(LA)_8_ promoiety (black circles) leads to enhanced compatibility with the hydrophobic core of PEG-*b*-PLA micelles and greater payload stability. Following release, o(LA)_8_-DTX prodrug is converted to DTX (blue hexagon) through intramolecular backbiting or cleavage via plasma esterases (yellow crescent shape). (**b**) In vivo tumor xenograft images for PC3 and LNCaP models treated with vehicle, Taxotere^®^, or o(LA)_8_-DTX-loaded micelles. (**c**) Corresponding tumor weight data show significantly improved tumor growth inhibition by o(LA)_8_-DTX micelles. (* *p* < 0.05, *** *p*  <  0.001). Reproduced with permission from [87]. Copyright 2021 Multidisciplinary Digital Publishing Institute (MDPI). (**B**) Design and evaluation of pH-responsive mPEG-pH-PCL micelles co-loaded with paclitaxel (PTX), etoposide (ETP), and rapamycin (RAPA). (**a**) Schematic diagram illustrating acid-triggered drug release after micellar endocytosis and endosomal trafficking. (**b**–**d**) Release profiles of PTX, ETP, and RAPA from micelles at varying pH values (7.4, 6.5, and 5.5). Reproduced with permission from [32]. Copyright 2023 Multidisciplinary Digital Publishing Institute (MDPI). (**C**) mPEG-PDLLA-PLL-based docetaxel micelles for breast cancer therapy. (**a**) Illustration of micelle formation and passive targeting through the EPR effect. The DTX-loaded micelles consist of a hydrophobic mPEG_2k_PDLLA_4k_ core and a PLL_1k_ hydrophilic shell. (**b**) Stability of DTX in micelle solution over time. In vitro release profiles of DTX in (**c**) physiological and (**d**) acidic pH conditions. (**e**) Tumor growth curves of different treatment groups. (* *p* < 0.05, ** *p* < 0.01). Reproduced with permission from [88]. Copyright 2017 Ivyspring International Publisher.

**Figure 7:**
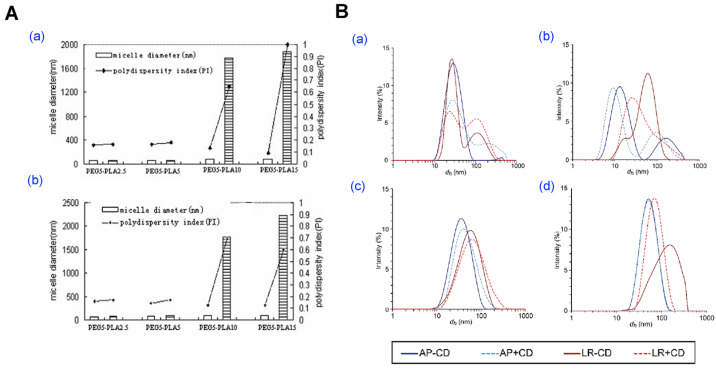
Preparation and characterization of polymeric micelles using the lyophilization method. (**A**) Evaluation of mPEG-PLA micelle stability before and after freeze–thaw and freeze–drying processes. (**a**,**b**) Micelle diameter and PDI of blank and AmB-loaded micelles before (white bars) and after freeze-drying (striped bars). Reproduced with permission from [96]. Copyright 2007 Wiley Periodicals, Inc. (**B**) Structural and aggregation behavior of PEG–PCL micelles following lyophilization and reconstitution. Particle size distribution (intensity) of (**a**) PEG_2_PCL_0.4_, (**b**) PEG_2_PCL_1.1_, (**c**) PEG_2_PCL_1.8_, and (**d**) PEG_2_PCL_4.0_ micelle prepared fresh (AP), after lyophilization/reconstitution (LR), and in the presence or absence of β-cyclodextrin (± CD). Reproduced with permission from [33]. Copyright 2023 Multidisciplinary Digital Publishing Institute (MDPI).

The development of polymeric micelles as drug delivery systems has historically relied on a set of foundational fabrication techniques based on the self-assembly of amphiphilic block copolymers in aqueous media. While these conventional methods have been widely utilized in academic and early translational research, they present limitations with respect to drug loading capacity, reproducibility, and scalability. This section reviews five principal fabrication approaches direct dissolution, dialysis, emulsification and solvent evaporation, thin-film hydration, and freeze-drying along with a comparative evaluation of their performance and practical applicability (Figure 3, Figure 4, Figure 5, Figure 6 and Figure 7).

### 3.1. Direct Dissolution

Direct dissolution is among the simplest and most accessible techniques for micelle preparation (Figure 3) [27,28,68,69,70,71,72,73,74]. The copolymer is dissolved in water or aqueous buffer, followed by the addition of the hydrophobic drug under agitation or mild heating. Micelle formation occurs spontaneously when the polymer concentration exceeds its critical micelle concentration (CMC), resulting in drug encapsulation within the hydrophobic core.

Several studies have demonstrated the feasibility of this method for encapsulating moderately hydrophobic drugs. For example, Mazzotta et al. successfully formulated caffeic acid-loaded P123 micelles, achieving improved aqueous solubility and light stability (Figure 3A) [28]. As a prodrug-enabled variant operationally analogous to direct dissolution, Duan et al. prepared a glutathione-responsive paclitaxel–GSSG–PEG conjugate that self-assembles into micelles upon aqueous dissolution without organic solvent, yielding particles of ~83 nm (DLS), stability in pH 7.4 PBS for 7 days, and GSH-triggered PTX release (drug content ~13% by NMR; cumulative release ~72% at 120 h in 5 mg mL^−1^ GSH) (Figure 3B) [75]. In addition, a hydrotrope-assisted direct dissolution route has been shown to eliminate organic solvents altogether: Huh et al. designed PEG-b-poly(4-(2-vinylbenzyloxy-N-picolylnicotinamide)) (PEG-b-P(2-VBOPNA)) block copolymers that solubilize paclitaxel directly in water and form micelles in a pH-dependent manner (no micellization at pH ≤ 2 due to pyridinium protonation; enhanced PTX solubility and micellization above pH 2). Longer hydrotropic blocks increased aqueous PTX solubility and slightly slowed release; under hydrotropic sink conditions, most PTX was released within 12 h, illustrating an organic-solvent-free direct dissolution pathway for highly hydrophobic APIs (Figure 3C) [74].

Despite its operational simplicity and solvent-free nature, this method is limited by poor encapsulation of highly hydrophobic drugs and low kinetic stability, which may result in premature drug leakage or aggregation. It is most suitable for preliminary formulation screening or for drugs with partial water solubility.

### 3.2. Dialysis

The dialysis method provides superior micelle uniformity and encapsulation stability by enabling thermodynamically driven self-assembly under controlled solvent exchange (Figure 4) [29,30,65,76,77,78,79,80,81]. The drug and copolymer are first dissolved in a water-miscible organic solvent (e.g., DMF, THF, or ethanol) and then placed in a dialysis membrane, which is immersed in water for 24–72 h.

Several experimental studies have validated its applicability for poorly water-soluble drugs. For instance, Yang et al. fabricated 10-hydroxycamptothecin-loaded PEG-*b*-PLA micelles via dialysis, demonstrating significantly enhanced cytotoxicity and faster drug release compared to PLA-only micelles, attributed to improved hydrophilic–hydrophobic phase separation and core–shell architecture (Figure 4A) [76]. Song et al. reported that curcumin-loaded PLGA-PEG-PLGA micelles prepared by dialysis exhibited prolonged plasma half-life (3.2 h vs. 0.8 h) and improved brain distribution in rats, suggesting the potential for systemic and CNS-targeted delivery (Figure 4B) [77]. In a separate study, Ozturk et al. prepared dorzolamide-loaded micelles using smart graft copolymers based on chitosan-*g*-PCL via dialysis, achieving high drug loading (~17%), prolonged release up to 6 days, and excellent in vitro biocompatibility, highlighting the potential for glaucoma therapy (Figure 4C) [78].

While dialysis yields uniform and stable micelles, it is labor-intensive, time-consuming, and difficult to scale. Residual solvents must be carefully removed, and membrane characteristics (e.g., pore size, material, etc.) can influence final particle attributes. Accordingly, its use is largely restricted to proof-of-concept studies.

### 3.3. Emulsification and Solvent Evaporation

This technique involves dissolving the copolymer and drug in a water-immiscible organic solvent, followed by emulsification into an aqueous phase through sonication or high-shear homogenization (Figure 5). Solvent evaporation under reduced pressure then induces micelle formation through polymer precipitation [31,82,83,84,85,86].

Several studies have utilized this approach to prepare stable micellar systems for hydrophobic drugs. Wang et al. fabricated all-HPMA micelles using a dichloromethane–water system and solvent extraction, achieving precise control of particle size from 50 to 200 nm by adjusting polymer and surfactant concentrations (Figure 5A). While this study did not include drug loading, it demonstrated the structural tunability of the emulsification–evaporation approach, providing a foundation for future drug delivery applications [31]. In another study, Bagheri et al. employed the emulsification and solvent evaporation method to formulate mPEG-*b*-p(HPMA-Bz) micelles. The polymer was dissolved in dichloromethane and emulsified into an aqueous solution containing polysorbate 80. After solvent removal, the resulting micelles exhibited sizes tunable from approximately 25 to 100 nm, depending on formulation variables such as polymer concentration, aqueous-to-organic phase ratio, and surfactant amount (Figure 5B) [82]. Lastly, Wang et al. developed Soluplus^®^ micelles loaded with FLQY2 (7-p-trifluoromethylphenyl-FL118), a camptothecin analog, using dichloromethane emulsification and solvent evaporation (Figure 5C). The system exhibited a mean size of 153 nm, high encapsulation efficiency (84.1%), and a 12.3-fold increase in oral bioavailability compared to a conventional HP-β-CD formulation [83].

Despite its effectiveness for hydrophobic drugs, this method requires rigorous removal of toxic solvents (e.g., chloroform, dichloromethane) and precise control of formulation parameters, including emulsification shear, solvent evaporation rate, and surfactant concentration. These complexities hinder scalability and necessitate real-time analytical monitoring for process standardization.

### 3.4. Thin-Film Hydration

In thin-film hydration, the copolymer and drug are co-dissolved in a volatile solvent, which is evaporated to form a dry polymer film (Figure 6) [32,52,87,88,89,90,91,92,93,94]. Hydration of the film with aqueous buffer under agitation or sonication facilitates micelle self-assembly.

For example, Repp et al. employed thin-film hydration to prepare PEG-b-PLA micelles loaded with an oligo(lactic acid)_8_-docetaxel prodrug, achieving 50% (*w*/*w*) loading and prolonged drug retention in vivo (Figure 6A) [87]. Jo et al. utilized citraconic amide-linked mPEG-pH-PCL copolymers to fabricate pH-sensitive micelles co-encapsulating paclitaxel, etoposide, and rapamycin, enabling selective drug release in acidic tumor environments (Figure 6B) [32]. Tan et al. developed mPEG-PDLLA-PLL triblock copolymer micelles with enhanced docetaxel loading (20%), which improved anticancer efficacy and stability, attributed to compact pH-dependent drug release and drug–polymer interactions confirmed via molecular simulations (Figure 6C) [88].

This technique allows for concentrated dispersions and broad polymer compatibility, but its reproducibility depends on tight control of film thickness, hydration conditions, and drying rate. Additional sonication or rehydration steps are often necessary, introducing variability during scale-up.

### 3.5. Freeze-Drying (Lyophilization)

Freeze-drying is used both as a post-processing step for micelle stabilization and, in some cases, as a direct preparation method when employing aqueous co-solvent systems (e.g., tert-butanol/water), allowing for prolonged micelle storage and preventing drug degradation during storage (Figure 7). Upon rehydration, micelles can reform if their structure is preserved during drying [33,95,96].

Notably, Yang et al. demonstrated that amphotericin B-loaded PEG–PLA micelles retained their spherical morphology and redispersion ability when lyophilized with polyethylene glycol (PEG) as a cryoprotectant, particularly in copolymers with a higher PEG-to-PLA ratio. In contrast, micelles with higher PLA content exhibited poor redispersibility and aggregation after drying, underscoring the critical role of hydrophilic segment density and steric stabilization in successful freeze-drying outcomes (Figure 7A) [96]. Likewise, Hussain et al. reported that β-cyclodextrin significantly improved the redispersibility of both blank and drug-loaded PEG-*b*-PCL micelles after lyophilization, whereas formulations without cryoprotectants resulted in poor recovery and morphological collapse (Figure 7B) [33].

Although freeze-drying enhances shelf stability and simplifies transport, it presents formulation challenges including drug recrystallization, excipient incompatibility, and incomplete micelle recovery. Optimizing lyophilization parameters and selecting suitable stabilizers are critical for maintaining micelle integrity.

### 3.6. Comparative Evaluation of Conventional Methods

Conventional fabrication methods provide essential tools for early-stage formulation development, offering operational simplicity and broad applicability. However, each technique presents trade-offs (Table 1). Direct dissolution is limited by low drug loading and poor stability [28,71,78]. Dialysis provides excellent uniformity but lacks scalability [29,30,78]. Emulsification supports high drug loading but poses reproducibility and toxicity concerns [31,82,83]. Thin-film hydration is versatile but sensitive to process variables, while freeze-drying enables long-term storage yet requires careful optimization [33,52,91].

To enable like-for-like comparisons under comparable chemistries, we compiled PEG–PLA micelles prepared by representative conventional methods and abstracted three quality attributes including hydrodynamic size (DLS, nm), polydispersity (PDI), and drug-loading content (DL, % *w*/*w*) into Table 2. For consistency, DL is reported as loaded drug/(loaded drug + polymer) × 100, whereas encapsulation efficiency (EE, % of input drug encapsulated) is not converted to DL when only EE was available (left as NR; see Table 2 caption). As summarized in Table 2, thin-film hydration typically yields small, narrowly dispersed micelles with mid-range DL; dialysis/co-solvent exchange tends to produce larger and broader distributions with lower DL; emulsification–solvent evaporation gives intermediate sizes with method-dependent DL; and API loading followed by lyophilization preserves size/PDI upon reconstitution, supporting manufacturability. These quantitative trends complement the process-level indicators (residual-solvent control, throughput, and readiness for continuous processing and PAT) referenced throughout this review in Table 3.

Building on the quantitative comparisons in Table 1 and Table 2 these limitations have driven the exploration of next-generation micelle fabrication strategies designed to enhance process control, eliminate toxic solvents, and support industrial-scale manufacturing. The following section examines these emerging approaches including microfluidics, supercritical fluids, stimuli-responsive systems, and PEG-assisted methods, which collectively represent a paradigm shift toward robust and pharmaceutically viable micelle technologies.

## 4. Emerging and Scalable Preparation Strategies

**Figure 8:**
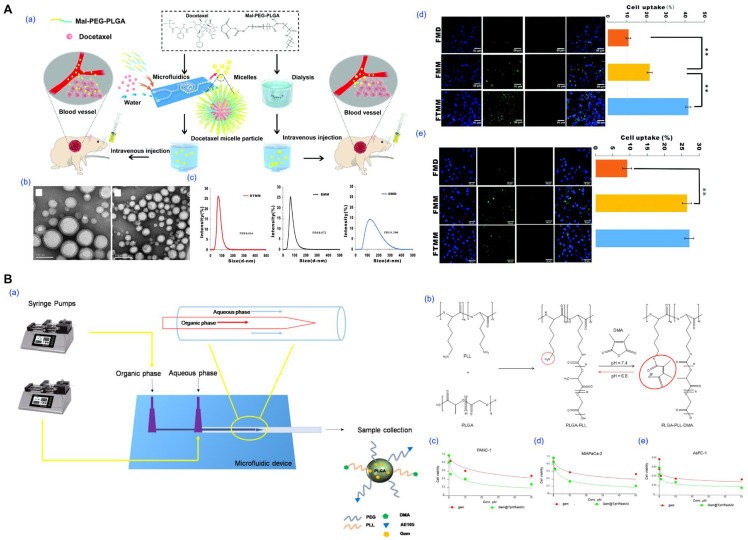
Representative examples of polymeric micelle preparation using microfluidic-assisted methods. (**A**) Docetaxel-loaded micelles were fabricated using PLGA-PEG-Mal block copolymers via a microfluidic mixing platform, followed by dialysis to remove organic solvent residues. (**a**) Schematic overview of the formulation process comparing microfluidic versus bulk preparation methods and subsequent intravenous administration in tumor-bearing mice. (**b**) Transmission electron microscopy (TEM) images of microfluidics-prepared micelles. (**c**) Size distribution and polydispersity index (PDI) of various micelle formulations (DTMM, DMM, DMD). (**d**,**e**) Cellular uptake of micelles in A549 and 3LL cancer cells was analyzed by confocal fluorescence microscopy and flow cytometry after 6 h incubation. (** *p* < 0.01). Reproduced with permission from [34]. Copyright 2018 The Royal Society of Chemistry. (**B**) Development of pH-sensitive micellar systems using a microfluidic mixing approach. (**a**) Schematic representation of the microfluidic setup used to co-assemble PLGA-PEG and PLGA-PLL-DMA copolymers into mixed micelles under controlled flow conditions. (**b**) Synthetic route of the PLGA-PLL-DMA polymer. In vitro cytotoxicity of free gemcitabine and gem@TpHResMic micelles against (**c**) PANC-1, (**d**) MIA PaCa-2, (**e**) AsPC-1. Reproduced with permission from [38]. Copyright 2021 Multidisciplinary Digital Publishing Institute (MDPI).

**Figure 9:**
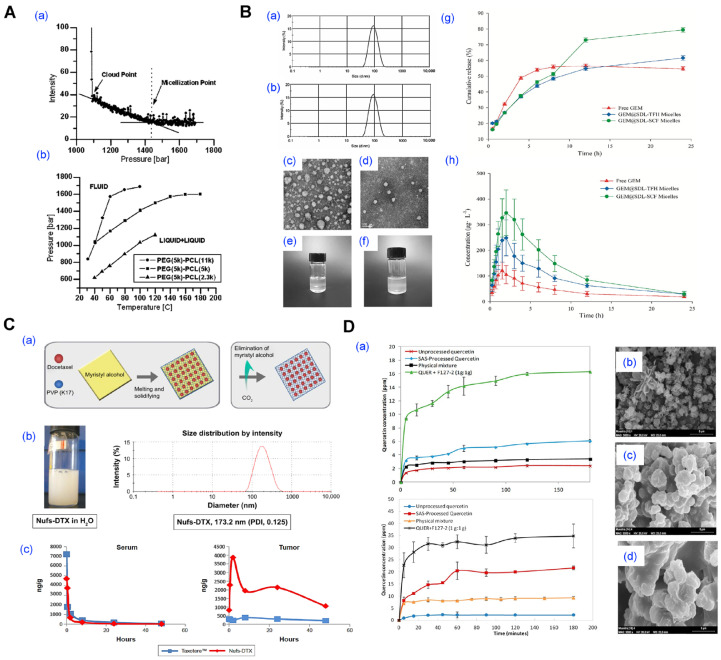
Supercritical fluid (SCF)-based micelle fabrication strategies and their physicochemical characterization. (**A**) Phase behavior and micellization profiles of PEG-*b*-PCL block copolymers in halogenated supercritical solvents. (**a**) Light scattering intensity profile of PEG-*b*-PCL (5–2.3 k) in supercritical trifluoromethane at 100 °C. (**b**) Expanded P–T diagrams comparing the block copolymer with various hydrophobic segment lengths (PEG-*b*-PCL 5–11 k, 5–5 k, and 5–2.3 k). Reproduced with permission from [35]. Copyright 2009 Springer Science + Business Media, LLC. (**B**) Characterization of GEM-loaded micelles prepared by thin-film hydration (TFH) and SCF-assisted methods. (**a**,**b**) Particle size distribution, (**c**,**d**) TEM micrographs, and (**e**,**f**) photographs of GEM@SDL-TFH and GEM@SDL-SCF micelles, respectively. (**g**) In vitro drug release kinetics of free GEM and both micelle types over 24 h. (**h**) Plasma concentration–time curves following oral administration of 20 mg·kg^−1^ GEM, GEM@SDL-TFH, and GEM@SDL-SCF micelles in rats. Reproduced with permission from [39]. Copyright 2019 Elsevier. (**C**) Formation of docetaxel nanoparticles (Nufs-DTX) using a supercritical fluid–fat extraction technique. (**a**) Schematic of formulation strategy utilizing myristyl alcohol as a dispersion medium for docetaxel and PVP, followed by CO_2_-mediated extraction. (**b**) Photograph of Nufs-DTX dispersion in water and corresponding size distribution. (**c**) Tumor accumulation and retention of Nufs-DTX. Reproduced with permission from [109]. Copyright 2015 Dovepress. (**D**) SAS (supercritical antisolvent) process for the preparation of Pluronic-based micellar particles. (**a**) The dissolution and solubility of the QUER + F127-2 formulation. (**b**–**d**) SEM images of particles produced via SAS co-precipitation of Pluronic F127 and quercetin. Reproduced with permission from [110]. Copyright 2014 American Chemical Society.

**Figure 10:**
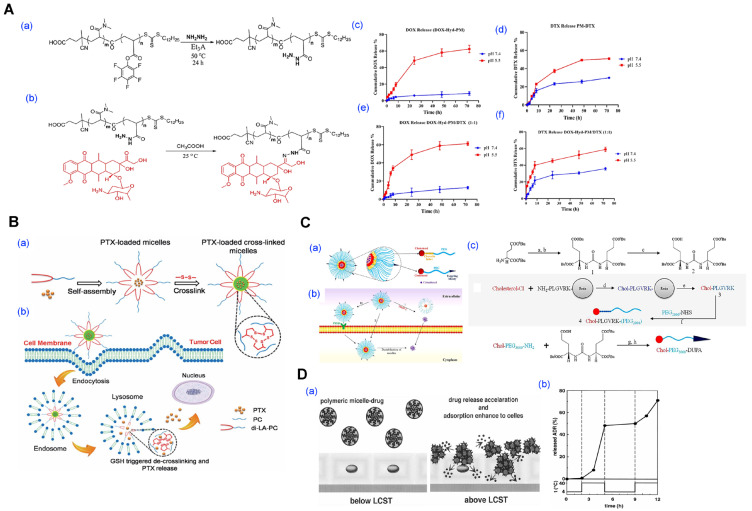
Representative examples of stimuli-triggered micelle formation and controlled drug release. (**A**) Dual drug-loaded micelles with pH-responsive hydrazone linkers for sequential release. (**a**,**b**) Synthetic schemes illustrating the conjugation of doxorubicin (DOX) and docetaxel (DTX) to the p(DMA-co-PFPA) backbone via hydrazone linkages. In vitro release profiles of (**c**,**e**) DOX and (**d**,**f**) DTX from single or combination-loaded micelles at pH 7.4 and 5.5. Reproduced with permission from [36]. Copyright 2024 BioMed Central (BMC). (**B**) Reduction-responsive crosslinked micelles based on di-LA-PC for glutathione (GSH)-triggered release of paclitaxel (PTX). (**a**) Schematic of micelle formation and crosslinking via disulfide bonds, followed by intracellular reduction-triggered disruption. (**b**) Cellular uptake and micelle disassembly mechanisms. Reproduced with permission from [40]. Copyright 2021 The Royal Society of Chemistry. (**C**) Enzyme-responsive micelles for prostate cancer targeting. (**a**,**b**) Schematic of micelle self-assembly and enzyme-mediated disassembly via MMP-2 cleavage. (**c**) Synthetic pathways for DUPA and cholesterol-PEG derivatives used for targeted delivery. Reproduced with permission from [64]. Copyright 2020 Elsevier. (**D**) Thermo-responsive micelles for temperature-controlled drug delivery. (**a**) Phase transition of PIPAAm–PBMA micelles below and above the lower critical solution temperature (LCST). (**b**) Controlled release and on/off switching of ADR release based on thermal stimuli. Reproduced with permission from [62]. Copyright 1999 Elsevier.

**Figure 11:**
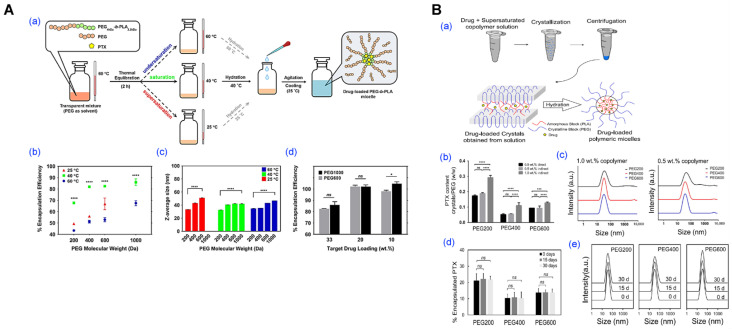
Representative examples of micelle fabrication via PEG-assist and crystallization-from-supersaturated-solution methods. (**A**) Fabrication of paclitaxel (PTX)-loaded polymeric micelles via PEG-assist method. (**a**) Schematic of the PEG-assisted preparation process, in which PEG of various molecular weights is used to solubilize copolymer and drug components, followed by hydration to induce micelle formation. (**b**,**c**) Effect of PEG molecular weight and hydration temperature on encapsulation efficiency and particle size, respectively, at a target drug loading of 33.0 wt%. (**d**) Encapsulation efficiency measured at various drug loadings under optimized conditions. (* p < 0.05, **** *p* < 0.0001, ns = non-significant). Reproduced with permission from [37]. Copyright 2022 Elsevier. (**B**) Micelle formation using crystallization-from-supersaturated-solution strategy. (**a**) Schematic representation of the crystallization-based micelle fabrication process involving drug–copolymer–PEG co-crystallization, separation, and hydration into nanomicelles. (**b**) PTX content retained in crystals versus PEG solution, and (**c**) corresponding particle size distribution. (**d**,**e**) Long-term stability of stored crystals and micelle formation efficiency after hydration. (*** *p* < 0.001, **** *p* < 0.0001, ns = non-significant). Reproduced with permission from [41]. Copyright 2025 Elsevier.

The limitations inherent in conventional polymeric micelle fabrication methods such as low drug loading efficiency, reproducibility issues, and scalability challenges have spurred the development of innovative strategies aimed at enhancing process control, eliminating toxic solvents, and facilitating industrial-scale production. This section explores four prominent emerging techniques: microfluidic assembly, supercritical fluid processing, stimuli-responsive systems, and PEG-assisted methods (Figure 8, Figure 9, Figure 10 and Figure 11).

### 4.1. Microfluidic-Assisted Fabrication

Microfluidic-assisted micelle fabrication provides a highly controlled environment for nanocarrier assembly by precisely manipulating laminar flow and solvent mixing kinetics within microscale channels (Figure 8) [34,38,99,111]. The core mechanism involves the rapid mixing of an organic phase containing the amphiphilic block copolymer and drug with an aqueous phase, typically in Y-junction or flow-focusing geometries. The diffusive interface formed between these streams facilitates controlled nanoprecipitation and polymer self-assembly, resulting in micelles with narrow size distributions and improved batch-to-batch reproducibility.

Bao et al. demonstrated the superiority of microfluidic preparation by fabricating docetaxel-loaded PLGA-PEG-Mal micelles with a mean size of 72 nm and low PDI (0.072), in contrast to dialysis-based micelles which exhibited broader size distribution and lower drug loading (Figure 8A) [34]. The microfluidic micelles achieved higher cytotoxicity in A549 cells and significantly improved tumor inhibition in vivo, with a suppression rate exceeding 90%. In another study, Iacobazzi et al. developed gemcitabine-loaded, pH-responsive, and uPAR-targeted micelles using microfluidic-assisted co-flow nanoprecipitation (Figure 8B) [38]. The resulting formulation selectively released the drug in acidic environments and exhibited enhanced cytotoxicity in both 2D and 3D pancreatic cancer models, as well as the ability to overcome cancer-associated fibroblast-mediated resistance.

These examples highlight the strengths of microfluidic approaches in enabling fine-tuned control over micelle characteristics and functional design, offering a versatile platform for next-generation drug delivery systems.

### 4.2. Supercritical Fluid Processing

Supercritical fluid (SCF) processing has emerged as a solvent-minimized or solvent-free approach for preparing polymeric micelles and other nanocarriers (Figure 9) [35,39,109,110]. This technique exploits the unique physicochemical properties of SCFs, particularly carbon dioxide (scCO_2_) and trifluoromethane, which exhibit liquid-like solvation and gas-like diffusivity above their critical temperature and pressure. These characteristics enable controlled precipitation, solvent extraction, or phase inversion for micelle formation either during SCF exposure or after rehydration of a dried matrix.

For example, Tyrrell et al. demonstrated that PEG-*b*-PCL block copolymers form stable micellar nanoparticles in supercritical trifluoromethane, which, upon rapid depressurization, yield dry powders that spontaneously reassemble into micelles in water without the use of organic solvents (Figure 9A) [35]. In another study, Hou et al. prepared Soluplus/DSPE-PEG2000-based micelles loaded with germacrone using an improved SCF-assisted reverse phase evaporation method, achieving significantly smaller particle sizes, enhanced drug loading, and a 298% increase in oral bioavailability compared to conventional thin-film hydration methods (Figure 9B) [39]. Choi et al. applied a proprietary NUFS™ process, utilizing SCF extraction of fatty excipients, to generate carrier-minimized docetaxel nanoparticles with superior tumor accumulation and reduced toxicity versus commercial Taxotere™, underscoring the potential of SCF methods for clinical translation (Figure 9C) [109]. Similarly, Fraile et al. employed the supercritical antisolvent (SAS) method to co-precipitate quercetin with Pluronic F127, producing amorphous micellar formulations with improved aqueous solubility and dissolution rates in simulated gastric and intestinal fluids (Figure 9D) [110].

SCF processing offers advantages such as avoiding toxic solvents, enabling low-temperature conditions for thermolabile drugs, and producing dry micellar powders suited for long-term storage. However, its implementation is limited by the need for high-pressure equipment, specialized formulation development, and narrow process windows that require precise control of temperature, pressure, and flow rate. These factors increase cost and complexity and pose challenges for GMP (Good Manufacturing Practice) standardization and scalability.

### 4.3. Stimuli-Triggered Micelle Formation

Stimuli-triggered micelle formation is an advanced strategy in nanocarrier engineering, where self-assembly is initiated or modulated by specific environmental cues such as pH, redox potential, enzymatic activity, or temperature (Figure 10) [1,36,40,62,63,64,65,112]. Unlike conventional micelles that assemble spontaneously in aqueous media, these systems form or disassemble selectively in pathological or intracellular environments, providing spatial and temporal control over drug release. While many stimuli-responsive systems focus on triggered release from preformed micelles, recent approaches leverage stimulus-induced micelle formation itself to enhance specificity and minimize systemic exposure.

For instance, Farhoudi et al. developed a pH-sensitive polymeric micelle system in which doxorubicin was covalently conjugated to the polymer via a hydrazone linkage, and docetaxel was physically loaded into the hydrophobic core (Figure 10A) [36]. The micelles remained stable under physiological pH but disassembled in mildly acidic tumor environments (pH 5.5), enabling dual-drug release and achieving a synergistic cytotoxic effect in both in vitro and in vivo breast cancer models. Similarly, Li and colleagues introduced a redox-responsive micellar system based on a small amphiphilic molecule, di-lipoyl-glycerophosphorylcholine, which underwent disulfide crosslinking to ensure stability in circulation (Figure 10B) [40]. Upon exposure to intracellular glutathione-rich conditions, the crosslinks cleaved, triggering micelle disassembly and rapid paclitaxel release at the tumor site. In another approach, Barve et al. constructed enzyme-responsive micelles by integrating matrix metalloproteinase-2 (MMP-2) cleavable peptide linkers between the hydrophilic PEG and hydrophobic cholesterol segments (Figure 10C) [64]. These micelles remained intact during systemic circulation but dissociated selectively in the MMP-2-rich prostate tumor microenvironment, thereby enhancing the accumulation and efficacy of cabazitaxel in vivo. Thermo-responsive micelle formation has also been exploited. Chung et al. utilized poly(N-isopropylacrylamide)-*b*-poly(butylmethacrylate) to form micelles that remained stable below the lower critical solution temperature (LCST), but collapsed and released adriamycin rapidly upon mild hyperthermia, demonstrating a clear temperature-dependent drug release profile (Figure 10D) [62].

These systems enable precise drug release and enhanced therapeutic index. However, they present challenges in synthesis (multi-step reactions, structural precision), variable trigger responsiveness across patient populations, and scale-up under environmentally stable but responsive conditions. Furthermore, regulatory pathways for such complex, adaptive systems are not yet well defined, requiring extensive characterization.

### 4.4. PEG-Assisted Method

The PEG-assisted method has gained recognition as a practical and scalable approach for polymeric micelle fabrication, owing to its simplicity, solvent-free operation, and compatibility with pharmaceutical manufacturing standards (Figure 11) [37]. In this technique, amphiphilic block copolymers and hydrophobic drugs are co-dissolved in low-molecular-weight PEGs (e.g., PEG 1000 or 2000), followed by gradual hydration under mild conditions. This enables spontaneous micellization without dialysis, volatile solvents, or mechanical emulsification, streamlining early formulation screening and facilitating GMP-compliant production.

A key advantage lies in the multifunctional role of PEG, as a solvent, stabilizer, and plasticizer, which promotes efficient self-assembly while maintaining high biocompatibility. Its regulatory approval across multiple administration routes (parenteral, oral, topical) facilitates rapid translation into clinical development without the need for additional safety validation [37]. Furthermore, this method is compatible with standard pharmaceutical equipment and freeze-drying, offering exceptional stability and formulation flexibility.

Rasoulianboroujeni et al. successfully formulated paclitaxel-loaded mPEG-*b*-PLA micelles by dispersing the polymer–drug mixture in low molecular weight PEGs such as PEG 1000, followed by hydration at room temperature (Figure 11A) [37]. The resulting micelles demonstrated high encapsulation efficiency (~85%), sub-100 nm particle size, and excellent colloidal stability, withstanding freeze-drying and rehydration without the need for cryoprotectants. In subsequent work, the same research group advanced this method by eliminating the need for freeze-drying for long-term storage and developed a new approach based on the crystallization of a supersaturated copolymer–drug solution. This method was applied for the encapsulation of both parent drugs and oligolactic acid prodrugs of paclitaxel, rapamycin, and docetaxel (Figure 11B) [41].

While certain formulations may require mild heating during the dispersion step, and a degree of pre-screening is advisable for drugs with extremely low PEG compatibility, these are minor technical considerations that do not detract from the method’s overall appeal. In fact, its operational simplicity, minimal equipment requirements, and elimination of volatile or toxic solvents make it especially attractive for laboratories and manufacturing settings aiming to accelerate formulation development without compromising reproducibility or safety. Moreover, the process is highly reproducible and readily scalable, lending itself well to both small-batch prototyping and large-scale production within GMP frameworks.

Importantly, the PEG-assisted method is particularly well-suited for drugs with moderate to high PEG solubility, a category that includes a wide range of hydrophobic small molecules currently under clinical and preclinical investigation. For such compounds, this method enables rapid micelle formation with high encapsulation efficiency, excellent colloidal stability, and compatibility with downstream processing steps such as sterile filtration and lyophilization. These features collectively position the PEG-assisted approach not merely as an alternative to traditional micellization techniques, but as a strategically advantageous platform—one that offers a streamlined, solvent-free, and regulatorily favorable pathway for the development of nanomedicines optimized for clinical translation and commercial deployment.

### 4.5. Comparative Analysis and Scale-Up Considerations

The growing diversity of micelle fabrication strategies such as microfluidic-assisted assembly, supercritical fluid (SCF) processing, stimuli-triggered micellization, and PEG-assisted methods reflects a broader shift toward improved process control, reduced solvent use, and better regulatory alignment (Table 4). Although these methods address limitations of conventional techniques, they vary in formulation flexibility, scalability, and industrial feasibility. Therefore, selecting an appropriate platform requires critical comparison based on intended application. As in Section 3.6, we adopt a like-for-like perspective at constant polymer/API; Table 2 lists similar polymer structure (mostly PEG–PLA) and taxane exemplars prepared via these emerging routes.

Microfluidic systems provide precise control over particle size and morphology via laminar flow and rapid mixing, enabling high reproducibility and early-stage optimization [34,38,99,111]. Yet, their industrial scalability remains limited due to low throughput, solvent compatibility issues, and the complexity of parallelization or continuous manufacturing integration. For scale-up, numbering-up with matched manifolds and at-/in-line PAT (e.g., DLS and UV–HPLC at the outlet) improves batch comparability while managing fouling and pressure drop. SCF processing allows solvent-minimized micelle formation under mild conditions, making it attractive for thermosensitive drugs [35,39,109,110]. Its ability to yield dry powders without residual solvents is a key advantage. However, the need for high-pressure equipment and process optimization restricts its use to niche, high-value products. A powder-first path can simplify fill-finish after reconstitution and reduce residual-solvent risk, but requires validated high-pressure safety, closed-loop CO_2_ recovery, and demonstration that reconstituted dispersions meet micelle CQAs (size/PDI, dilution stability). Stimuli-responsive micelles add environmental sensitivity such as pH or enzyme-triggered release, offering potential for targeted therapy, especially in oncology [36,40,62,63,64,65]. However, issues such as batch variability, trigger inconsistency across patients, and regulatory uncertainty limit their broader application. CMC-readiness hinges on linker integrity (orthogonal LC–MS), definition of clinically relevant trigger conditions for release testing, and mapping patient-to-patient variability in trigger strength.

PEG-assisted methods stand out for their simplicity, scalability, and regulatory compatibility [37]. Using pharmaceutically accepted PEG excipients, this approach avoids harmful solvents, supports lyophilization, and aligns well with GMP production. Its limitations are mainly tied to drug–PEG solubility compatibility and hydration control [41]. Unit operations (hydration with controlled kinetics, static/inline mixing, lyophilization) are readily implemented in GMP; key CPPs include PEG grade and water content, PEG:drug and PEG:polymer ratios, and the hydration temperature/rate.

Method-wise trade-offs are summarized in Table 3 using harmonized definitions (Size/PDI, DL/EE), manufacturability axes (scalability, solvent burden), and control enablers (PAT/QbD). No single method is universally optimal. Microfluidics offers precision for small-scale screening; SCF suits solvent-sensitive drugs; stimuli-responsive systems enhance biological selectivity; and PEG-assisted methods balance manufacturability and performance. Practical implementation will depend on drug properties, delivery route, and production infrastructure. Future progress will require integration of these methods into scalable, quality-controlled workflows. Adoption of QbD principles, in-line analytics, and modular platforms will be critical for advancing micelle-based therapeutics to market. Translation is accelerated by QbD-driven control strategies, PAT-enabled monitoring, and predefined comparability criteria when moving between platforms.

## 5. Applications of Polymeric Micelles in Drug Delivery

**Figure 12:**
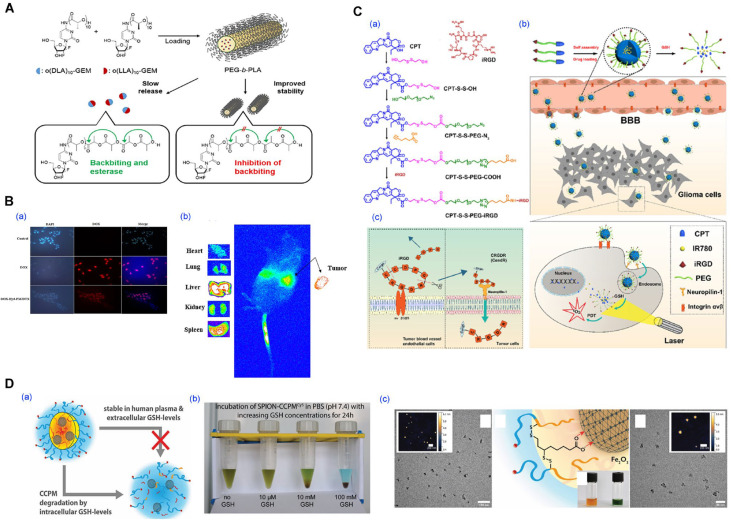
Polymeric micelle-based systems for anticancer drug delivery and therapeutic applications. (**A**) oligolactic acid conjugated-prodrug loaded micelle. Stereocomplex prodrugs of gemcitabine (GEM) synthesized using enantiomeric oligolactic acid chains (o(LLA)_n_-GEM and o(DLA)_n_-GEM. Reproduced with permission from [113]. Copyright 2018 American Chemical Society. (**B**) Evaluation of in vitro in vivo anticancer activity of dual drug-loaded micelles. (**a**) Fluorescence microscopy of 4T1 cells after treatment with free DOX or combination micelles (DOX-Hyd-PM/DTX). (**b**) Biodistribution imaging (in vivo and ex vivo) of DOX-Hyd-PM/DTX micelles after 24 h. Reproduced with permission from [36]. Copyright 2024 BioMed Central (BMC). (**C**) Blood–brain barrier (BBB)-penetrating micelles for glioma-targeted therapy. (**a**) Chemical structures and synthesis of disulfide-bridged camptothecin (CPT)-based polymers conjugated with iRGD for active targeting. (**b**) Schematic illustration of the mechanism. (**c**) Mechanistic representation of iRGD peptide’s dual role in BBB penetration and tumor-specific uptake. Reproduced with permission from [44]. Copyright 2020 Elsevier. (**D**) Diagnostics and responsive therapeutic micelle system. (**a**) Conceptual schematic of macrophage-internalized micelles and intracellular iron release. (**b**) GSH-triggered degradation of micelles in physiological buffer (pH 7.4, 37 °C). (**c**) SPION-core crosslinked micelles characterized by AFM and TEM imaging, with dye-labeling and stability confirmed in aqueous media. Reproduced with permission from [48]. Copyright 2021 Wiley-VCH.

**Figure 13:**
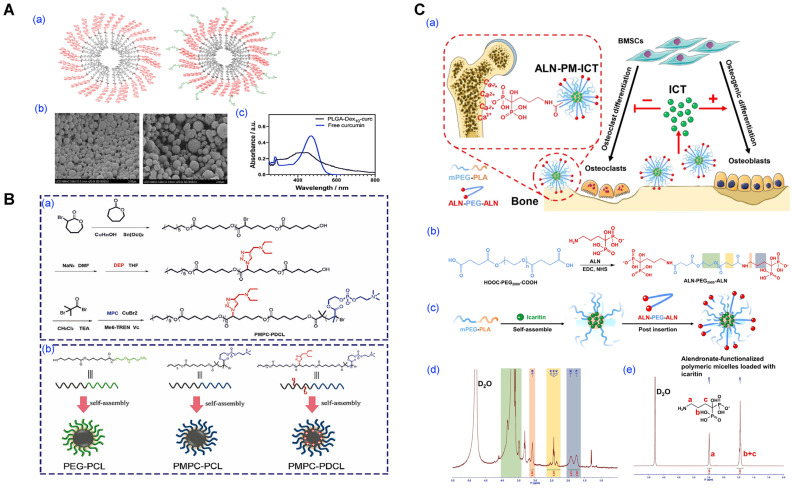
Polymeric micelles for antibacterial applications. (**A**) PLGA-based micelles loaded with curcumin for antibacterial. (**a**) Schematic representation of the micelle architecture before and after curcumin encapsulation. (**b**) SEM images comparing blank PLGA-Dex10 micelles and curcumin-loaded PLGA-Dex10-curc micelles. (**c**) UV–Vis absorption spectra showing the spectral differences between free curcumin in aqueous solution and curcumin encapsulated within PLGA-Dex10 micelles. Reproduced with permission from [125]. Copyright 2021 BioMed Central (BMC). (**B**) Design and self-assembly of zwitterionic polymeric micelles for enhanced antibiofilm performance. (**a**) Synthetic route for PMPC-PDCL amphiphilic block copolymer. (**b**) Self-assembly behavior of PEG-PCL, PMPC-PCL, and PMPC-PDCL block copolymers into micelles. Reproduced with permission from [119]. Copyright 2022 Wiley-VCH. (**C**) Alendronate-functionalized polymeric micelles for bone delivery (**a**) Schematic representation of the alendronate-functionalized polymeric micelles for bone-targeting delivery. (**b**) Scheme illustrating conjugation of alendronate to both sides of polyethylene glycol to generate polymeric micelles (**c**) Scheme illustrating preparation of alendronate-functionalized polymeric micelles loaded with icaritin. ^1^H NMR spectra of (**d**) the ALN-PEG-ALN conjugate and (**e**) free ALN in D_2_O (400 MHz). ALN, alendronate; PEG, polyethylene glycol; mPEG-PLA, methoxy poly(ethylene glycol)-poly(lactic acid). Reproduced with permission from [120]. Copyright 2024 Elsevier.

**Figure 14:**
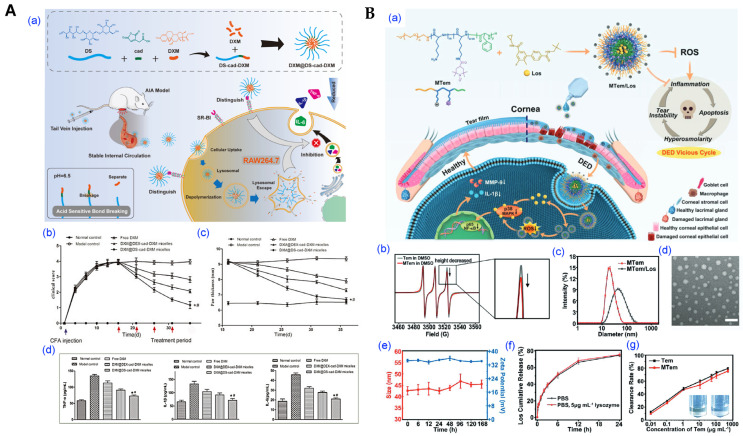
Polymeric micelles for anti-inflammatory applications. (**A**) Anti-inflammatory polymeric micelles for rheumatoid arthritis therapy. (**a**) Schematic of the design and administration of dexamethasone-loaded DS-cadherin micelles (DXM@DS-cad-DXM). (**b**,**c**) Clinical scores and paw thickness measured throughout the treatment period. (**d**) Quantitative analysis of pro-inflammatory cytokine levels in joint tissues. (* *p* < 0.05, # *p* < 0.05). Reproduced with permission from [129]. Copyright 2023 Multidisciplinary Digital Publishing Institute (MDPI). (**B**) Anti-oxidative and anti-Inflammatory micelles for dry eye. (**a**) Schematic route of synthesized MTem/Los and targeting mechanisms. (**b**) X-band EPR spectra of free Tem and MTem. (**c**) Size distribution of MTem and MTem/Los micelles. (**d**) TEM image of MTem/Los micelles. (**e**) Variation in hydrodynamic diameter and zeta potential of MTem micelles for Stability evaluation. (**f**) In vitro release profile of Los from MTem/Los micelles under PBS and PBS + lysozyme conditions. (**g**) Antioxidant capacity Tem and MTem micelles. Reproduced with permission from [132]. Copyright 2022 Wiley-VCH.

The development of advanced micelle preparation strategies has expanded the clinical scope of polymeric micelles beyond solubilization vehicles to multifunctional drug delivery platforms. Their ability to encapsulate hydrophobic drugs, improve pharmacokinetics, and integrate targeting or responsive features has led to significant applications in various therapeutic areas and administration routes. This section highlights representative applications of polymeric micelles, focusing on oncology, infectious and inflammatory diseases, and route-specific design considerations (Figure 12, Figure 13 and Figure 14).

### 5.1. Anticancer Application

Oncology remains the most extensively explored domain for micelle-based drug delivery [34,87,113,114,115,116]. The intrinsic characteristics of tumors such as leaky vasculature and impaired lymphatic drainage enable passive targeting through the enhanced permeability and retention (EPR) effect. Polymeric micelles have leveraged this phenomenon to accumulate hydrophobic chemotherapeutics selectively in tumor tissue, while recent advances in functional design and fabrication have further improved therapeutic precision (Figure 12).

For instance, anticancer prodrug-loaded mPEG-*b*-PLA micelles have demonstrated improved pharmacokinetics, prolonged circulation, and enhanced cytotoxicity against prostate cancer cells in vitro and in vivo, leading to tumor growth inhibition and enhanced therapeutic efficacy (Figure 12A) [87,113]. The formulation exhibited strong compatibility with PEG-assisted fabrication, allowing for high-concentration dispersions and direct freeze-drying. Similarly, microfluidic fabrication of Taxane analog-loaded Mal-PEG-PLA micelles achieved better tumor accumulation and cytotoxicity in xenograft models compared to traditional dialysis methods, underscoring the relevance of preparation techniques to therapeutic efficacy [34]. Furthermore, micelles prepared via supercritical fluid drying retained structure and activity upon rehydration, with docetaxel formulations exhibiting significant tumor suppression in animal models, offering a solvent-free path to clinical-grade cytotoxic formulations [109].

Beyond single agents, polymeric micelles are increasingly used for combination therapy [36,52]. Dual-hydrophobic co-loading (e.g., taxanes with anthracyclines or flavonoids) and orthogonal designs that pair a core-loaded small molecule with a macromolecular cargo (siRNA/peptide via shell conjugation or micelleplex formation) have shown synergistic efficacy at reduced doses [41,117,118]. Critically, preparation choices govern performance: microfluidic co-precipitation tightens size and PDI for dual cargos and facilitates freeze-dryable formats; PEG-assisted, low-solvent assembly limits exposure of labile biomolecules; and core/shell crosslinking curbs burst release and dilution-induced disassembly. These method-level levers map directly onto the process indicators consolidated in Table 3 (residual-solvent control, throughput, readiness for continuous/PAT) and are consistent with the size/PDI/DL trends summarized in Table 2.

As specific implementations of these strategies, co-delivery strategies have been employed to address tumor heterogeneity and drug resistance (Figure 12B) [36]. Micelles simultaneously loaded with doxorubicin and luteolin achieved synergistic cytotoxicity and reduced systemic toxicity [52]. Sequential or pH-triggered release profiles were achieved by combining chemical conjugation with physical entrapment, enabling temporal control of exposure in the tumor microenvironment [36].

Stimuli-responsive systems, such as redox-cleavable camptothecin micelles conjugated with iRGD peptides, enabled blood–brain barrier penetration and selective drug release in glioma models (Figure 12C) [44]. Furthermore, theranostic micelles incorporating imaging moieties like indocyanine green or iron oxide nanoparticles allowed simultaneous tumor imaging and therapy, facilitating real-time treatment monitoring (Figure 12D) [48,53].

Taken together, these applications illustrate how fabrication choices (e.g., microfluidics, PEG-assisted loading, supercritical drying) translate into clinically relevant quality attributes such as size, PDI, DL, and residual-solvent control supporting personalized and targeted cancer therapy across multiple administration routes.

### 5.2. Anti-Infective Applications

Clinically relevant infectious niches impose distinct transport and release challenges, such as dense extracellular polymeric substances in biofilms, acidic and lipase-rich pockets, and mineralized bone surfaces [70]. Recent work shows that tailoring micelle chemistry and preparation can improve penetration and on-site release (Figure 13).

Curcumin-functionalized PLGA-dextran micelles exhibited intrinsic antibacterial and antibiofilm activity against *Pseudomonas* species by leveraging both the antimicrobial properties of curcumin and the biofilm-penetrating ability of the dextran shell. This system not only improved bacterial growth inhibition compared to free curcumin but also effectively disrupted established biofilms and reduced cell viability within the EPS matrix (Figure 13A). Tooth-binding micelles that present hydroxyapatite-affinitive ligands achieved robust retention and triclosan delivery against oral biofilms, reducing bacterial burden in vivo [70]. Similarly, zwitterionic poly(phosphorylcholine)-based micelles loaded with triclosan penetrated biofilms more effectively than PEG-based analogs and released drug under acidic/lipase conditions, leading to enhanced anti-biofilm activity (Figure 13B) [119].

Beyond mucosal surfaces, bone infections (osteomyelitis) present a mineral hydroxyapatite interface amenable to ligand-mediated targeting [120,121,122]. Alendronate- or tetracycline-decorated micelles have been reported to bind hydroxyapatite and concentrate payloads at infected bone; more recent studies combine bone-targeting with pH/redox-responsive release to address osteomyelitis models [123].

Representative anti-infective payloads extend beyond triclosan/ciprofloxacin. Vancomycin has been encapsulated in PEG–PCL-family micelles (including PEG–PCL–PEG triblocks), showing improved antibacterial potency versus free drug in vitro; amphotericin B micelles have repeatedly reduced fungal burden while mitigating hemolysis and systemic toxicity relative to conventional formulations [124]. Across these studies, reported hydrodynamic diameters are typically 40–150 nm with PDI ≤ 0.3, while drug loading varies widely (≈2–12 wt%) depending on core compatibility and method.

From a manufacturing standpoint, most reported systems use standard self-assembly (dialysis/solvent-exchange); solvent-free PEG-assist and continuous microfluidic processing offer routes to reduce residual-solvent risk and improve batch-to-batch reproducibility, which is advantageous for local delivery formats (cements, coatings, injectables).

### 5.3. Anti-Inflammatory Applications

Inflammation is an innate immune response triggered by stimuli such as wounds or pathogen invasion. It is a protective mechanism of the body that involves numerous mediators, including immune cells and blood vessels [126,127]. This inflammatory mechanism promotes tissue regeneration, removes damaged tissue, and restores homeostasis. Naturally inflammation within the body is an essential process for preventing internal damage and protecting the body from harmful stimuli. However, if excessive or chronic, it can become a major cause of various inflammatory diseases, such as heart disease, diabetes, neurodegenerative diseases, and inflammatory bowel disease.

Polymeric micelles have increasingly been applied to infectious and inflammatory diseases, where challenges such as poor solubility, toxicity, and localized delivery complicate conventional therapies [128,129,130,131]. Micelles provide a means of improving solubility, extending tissue residence time, and enhancing target specificity in these contexts (Figure 14).

For inflammation-targeted therapy, dextran sulfate–dexamethasone conjugate micelles were shown to selectively accumulate in arthritic joints via scavenger receptor-mediated uptake (Figure 14A) [129]. These systems effectively suppressed local cytokine production and demonstrated high biocompatibility, supporting their potential in chronic inflammatory diseases such as rheumatoid arthritis. Natural products with known therapeutic benefits but poor bioavailability such as quercetin have also been formulated into polymeric micelles, resulting in improved solubility, stability, and bioactivity in models of infection and inflammation [130].

Drug delivery to the eye is challenging because of its complex excretion mechanisms. To overcome these limitations, micelles are applied to the eye to address existing ocular drug delivery challenges and achieve effective ocular drug delivery [132,133]. Micelles can be applied for ocular drug delivery to increase mucosal adhesion, achieve sustained drug release, and enhance therapeutic efficacy. Therefore, polymer-based drug delivery systems have high potential for enhancing ocular bioavailability. For example, a cationic polypeptide micelle (MTem/Los) loaded with the MARK inhibitor Los and conjugated with the ROS scavenger Tempo was synthesized [132]. This reduced the production of inflammatory cytokines and inflammatory activity, demonstrating potent anti-inflammatory effects. Therefore, MTem/Los exhibits excellent biocompatibility and tolerability, making it a potential candidate for the simple and rapid treatment of diseases induced by oxidative stress and inflammation. These examples illustrate how micellar platforms extend beyond cancer to address delivery challenges in anti-inflammatory diseases.

### 5.4. Route-Specific Micellar Formulations

The versatility of polymeric micelles extends to a broad range of administration routes, including intravenous (IV), oral, transdermal, and inhalable delivery. Each route presents unique physiological barriers that necessitate specific design strategies and preparation techniques (Table 5).

Intravenous injection remains the most widely used route, particularly for oncology. Micelles prepared using core-crosslinking or PEG-assisted methods have shown prolonged circulation, improved tumor targeting, and reduced off-target toxicity [37,42]. FRET (Förster Resonance Energy Transfer)-based studies have confirmed the stability of PEG-*b*-PDLLA micelles in blood circulation and their ability to release drugs upon cellular uptake [49].

For oral delivery, micelles must withstand gastric degradation and transport drugs across the intestinal epithelium. pH-sensitive micelles encapsulating camptothecin demonstrated stability in acidic environments and rapid release at neutral pH, leading to increased drug permeability compared with the free drugs, in a 3D intestinal cell-based model [47]. Transporter-targeted systems using carnitine ligands also improved the oral absorption of gemcitabine, while chitosan-based micelles enhanced paclitaxel bioavailability by inhibiting P-glycoprotein-mediated efflux [43,46].

Transdermal micelle systems have enabled non-invasive delivery of hydrophobic agents. Coenzyme Q10 and curcumin-loaded hyaluronan micelles accumulated in the dermis and exhibited prolonged skin retention [50]. Lecithin-based micelles containing thymoquinone accelerated wound healing, demonstrating their utility in dermatological and regenerative applications [45].

Inhalable micelles represent an emerging strategy for pulmonary delivery of drugs requiring local or systemic action. TPGS/DPPC mixed micelles for lung cancer and mannose-decorated Soluplus^®^ micelles for tuberculosis, enable efficient aerosol delivery with enhanced lung accumulation and therapeutic efficacy. These systems demonstrate improved cytotoxicity or antimicrobial activity compared to free drugs or non-targeted micelles. Their nanoscale size and targeting design underscore the promise of micelles in pulmonary drug delivery [51,54].

## 6. Clinical Potential, Challenges, and Future Perspectives

Polymeric micelles have progressed beyond their initial role as solubilization vehicles to become multifunctional nanocarriers with validated in vivo efficacy across various therapeutic areas. Clinically approved formulations such as Genexol^®^-PM and Nanoxel^®^-M, as well as investigational systems like NK105 and NC-6004, underscore their translational relevance in oncology. The expansion of micellar applications into anti-infective, inflammatory, and non-parenteral delivery routes further reflects their versatility. However, broad clinical adoption remains limited by challenges in scale-up reproducibility, formulation stability, and regulatory complexity with practical failure modes including lot-to-lot variability, incomplete redispersion after drying, and payload leakage under physiologic dilution that collectively erode potency and consistency.

A key translational bottleneck lies in the manufacturing scalability and batch consistency of micellar systems. Conventional methods such as dialysis and thin-film hydration, although effective at lab scale, often lead to variability in particle size, drug loading, and physical stability during scale-up due to changes in mixing history, membrane area-to-volume ratios, and solvent removal kinetics. Although emerging fabrication strategies including microfluidic-assisted mixing, supercritical fluid processing, and PEG-assisted self-assembly offer enhanced process control and GMP compatibility, in practice, each platform still has distinct boundaries that must be engineered into the control strategy. (i) microfluidics provides precise and narrow residence-time distributions but requires robust parallelization and solvent handling; (ii) supercritical-fluid (SCF) routes minimize residual solvent and can yield powder-first intermediates yet demand high-pressure assets and validated CO_2_ recovery; (iii) PEG-assisted self-assembly simplifies solvent handling and lyophilization but remains contingent on drug–PEG compatibility and hydration kinetics. Accordingly, no single method is universally optimal; platform selection should be performed at constant polymer/API with prespecified comparability criteria and clearly mapped critical process parameters (CPPs). Inline/atline monitoring (e.g., UV–HPLC for drug content, DLS for size/PDI) can be used to enforce end-pointing and reduce batch drift (Table 2).

Stability during storage and transport poses an additional hurdle. Aqueous micellar dispersions are inherently unstable due to drug leakage, hydrolysis, or microbial growth. Freeze-drying can improve stability but requires careful selection of cryoprotectants and reconstitution conditions. Beyond gross size/PDI, clinically meaningful stability should encompass redispersion time, leakage under sink-like dilution, and preservation of drug content after accelerated/long-term storage. Failure modes include ester hydrolysis of polyester cores, Ostwald ripening for partially crystalline payloads, and carrier–container interactions that deplete active content. Recent work employing crystallizable PEG matrices has demonstrated promising results in generating redispersible micellar powders without relying on traditional aqueous intermediate steps, offering solutions for long-term storage and cold chain independence provided that reconstitution restores all predefined critical quality attributes (CQAs) including hydrodynamic size/PDI windows, drug loading, leakage limits, and dilution stability within acceptance ranges.

On the regulatory front, micellar formulations, especially those incorporating targeting ligands, stimulus-responsive mechanisms, or multiple actives, are often categorized as complex products. This designation triggers additional regulatory scrutiny around CQAs, excipient safety, lot-to-lot reproducibility, and CMC documentation. Read-through from ICH Q8–Q12 suggests three practical imperatives: (i) define CQAs that tie directly to clinical performance (e.g., trigger specificity and leakage limits under physiologic dilution); (ii) identify critical material attributes (CMAs; polymer architecture, block lengths, PEG grade/water content, residual monomers) and CPPs (flow ratios/energy input, solvent composition, hydration temperature/rate, lyophilization cycle); and (iii) establish a comparability plan for platform transfers (e.g., microfluidic screening to scaled mixing; aqueous intermediates to powder-first fill–finish). Regulatory confidence hinges less on the choice of platform than on demonstrated process understanding, monitoring, and robust redispersion after stress.

To navigate these challenges, the field is increasingly embracing QbD principles and PAT. Integration of real-time monitoring, scalable formulation platforms, and data-driven optimization is becoming central to enabling reproducible, regulatory-compliant micelle production. Concretely, digital manufacturing stacks such as inline DLS and UV–HPLC at reactor outlets, feedback control of flow rates and solvent fractions, and electronic batch records (EBR) with automated deviation triggers can shrink lot variability and investigation time. Active-learning and multi-objective Bayesian optimization accelerate Design of Experiments (DoE) across polymer architecture, solids content, and drug-to-polymer ratios, while digital twins that link CMAs/CPPs to CQAs support tech transfer and change control. Moving beyond batch, continuous or semi-continuous implementations (e.g., numbered-up microreactors, T-/Y-mixers with controlled residence-time distributions, and closed-loop hydration–quench trains) provide liter-per-hour throughputs without sacrificing mixing control when paired with PAT based endpointing and fouling management. In this context, AI-assisted optimization reduces experimental burden and improves robustness: model predictive control (MPC) or reinforcement learning controllers can adjust flow rates and solvent fractions in real time to keep CQAs within specification under disturbances; transfer learning across APIs supports platform comparability and faster tech transfer; and uncertainty quantification from surrogate/digital-twin models should feed release criteria and change-control risk assessments to align with ICH Q8–Q12.

Looking forward, the future of micellar nanomedicine will be shaped by multi-domain convergence—where polymer chemistry, microfabrication, and regulatory science intersect. Personalized medicine approaches may benefit from companion diagnostics integrated within theranostic micelle systems. Likewise, non-invasive administration routes such as oral and transdermal delivery, supported by smart stimuli-responsive designs, will likely play an increasing role in broadening clinical applicability provided that trigger windows (pH, enzyme activity, redox) are tuned to patient variability and explicitly mapped into specifications and release testing. In parallel, clinically aligned supramolecular strategies such as crystallization-guided self-assembly and trigger-activated micellization offer levers to narrow size distributions and program release, facilitating platform comparability across sites.

In summary, despite remaining challenges, polymeric micelles offer a technically and clinically viable platform for next-generation drug delivery. Disciplined execution and continued innovation in formulation design, scalable manufacturing, and regulatory alignment will be critical to realizing their full therapeutic potential.

## 7. Conclusions

Polymeric micelles have emerged as a highly adaptable and clinically relevant drug delivery platform, offering distinct advantages in the solubilization, protection, and targeted delivery of bioactive compounds. Their hallmark core–shell structure and nanoscopic size enable enhanced pharmacokinetics and tissue specificity, while modular copolymer designs allow for fine-tuned control of stability, release kinetics, and biological interactions.

This review has outlined a comprehensive landscape of micelle preparation strategies, from traditional methods like direct dissolution and dialysis to advanced approaches including microfluidic-assisted fabrication, supercritical fluid processing, and PEG-assisted self-assembly. Each method offers distinct trade-offs in terms of scalability, reproducibility, and regulatory alignment, and their selection should be guided by the physicochemical properties of the drug, intended administration route, and manufacturing constraints.

Therapeutically, polymeric micelles have demonstrated broad utility in oncology, infectious disease, and inflammation, with applications extending across intravenous, oral, transdermal, and inhalable routes. Functional innovations such as co-delivery systems, stimulus-responsive release, and theranostic integration further extend their clinical value. Notably, recent advancements in PEG-assisted methods and crystallization-enabled micelle engineering have significantly improved scalability and formulation robustness, bringing micellar systems closer to clinical and commercial readiness.

Despite significant progress, challenges related to long-term stability, industrial-scale manufacturing, and complex regulatory classification remain. Bridging these gaps will require not only advances in formulation science but also the adoption of QbD frameworks, real-time analytics, and harmonized validation protocols. By aligning innovation with regulatory and practical imperatives, polymeric micelles are well-positioned to become a cornerstone technology in the next generation of nanomedicine.

## Figures and Tables

**Table 1:** Summary and comparison of five conventional fabrication methods for polymeric micelles, including their process features, strengths, limitations, and representative references. These methods remain foundational but present challenges in scalability and formulation reproducibility.

Methods	Key Characteristics	Advantages	Limitations	References
Direct Dissolution	Polymer and drug directly mixed in aqueous medium.	Simple, solvent-free, fast.	Low drug loading, poor stability for highly hydrophobic drugs.	[27,28,68,69,70,71,72,73,74,78]
Dialysis	Polymer-drug solution in organic solvent dialyzed into water.	Uniform particle size, stable micelles.	Labor-intensive, slow, not scalable.	[29,30,65,76,77,78,79,80,81,97]
Emulsification & Evaporation	Polymer and drug in organic solvent emulsified and solvent evaporated.	High drug loading, tunable size.	Toxic solvents used, batch variability, difficult scale-up.	[31,82,83,84,85,86,98]
Thin-Film Hydration	Polymer and drug dissolved in volatile solvent, film formed and hydrated.	Versatile, compatible with various drugs.	Sensitive to hydration/film parameters, needs rehydration optimization.	[32,52,87,88,89,90,91,92,93,94]
Freeze-Drying	Micelles lyophilized with or without cryoprotectants, rehydrated later.	Enhances shelf-life, useful post-processing method.	Risk of drug crystallization, collapse without cryoprotectants.	[33,95,96]

**Table 2:** Method-wise, like-for-like comparison of similar polymer-based micelles containing similar API (PTX or DTX) prepared by representative fabrication methods. Reported hydrodynamic size (DLS, nm), polydispersity (PDI), and drug loading content (DL, % *w*/*w* = m_drug_/(m_drug_ + m_polymer_) × 100) are transcribed from source papers; NR = not reported. When a source reported only encapsulation efficiency (EE, % of input drug encapsulated) we did not convert to DL and left DL as NR.

Methods	Polymer	API	Size (nm)	PDI	DL%	References
Direct Dissolution	PEG-PLA	PTX	178–276	0.15~0.19	30.6~52.2	[68]
	PEG-b–P(2-VBOPNA)	PTX	25–90	NR	NR	[74]
Dialysis	PEG-P(VBODENA)	PTX	105–120	NR	18.4–37.4	[97]
	PEG-PLA	PTX	NR	NR	20–28	[68]
Emulsification & Evaporation	PGG-DENA	PTX	~70	0.219	11.7	[98]
	PEG-PLA	DTX	~36.19	0.249	≤20%	[86]
Thin-Film Hydration	Genexol-PM (mPEG–PDLLA/PTX)	PTX	25–30	<0.1	16	[92]
	mPEG-PDLLA-Phe(Fmoc)	PTX	~45	0.112	NR	[93]
	PEG–PLA	PTX	25–104.5	<0.21	15–20	[94]
Freeze-Drying	PEG-PLA	DTX	~30.6	0.18	9.7	[87]
	PEG–PLA	Amphotericin B	50–91	<0.141	NR	[96]
Microfluidic-Assisted	Mal-PEG-PLA	DTX	72 ± 1	0.072	11.12 ± 1.17	[34]
	mPEG-b-p(HPMAm-Bz)	PTX	~80	<0.1	~80% (EE)	[99]
	PEG-PLA	PTX	15–70	0.18 ± 0.02	~20%	[100]
Supercritical Fluid (SCF)	Soluplus^®^, DSPE-PEG2000 and Lipoid S-75	GEM	86.3 ± 3.7	0.106 ± 0.008	5.93 ± 0.2	[39]
	PLLA-PEG-PLLA	PTX	~651	NR	18.1	[101]
Stimuli-Responsive	AG-PEG-SS-PLA	PTX	85 ± 2.5	NR	~8	[102]
	PLA–PEG–folate	DTX	~181	0.29	47.15	[103]
PEG-Assisted	PEG-PLA	PTX	~50	NR	~33	[37]
	PEG-PLA	PTX	~40	NR	~30	[41]

**Table 3:** Structured comparison of micelle fabrication methods.

Methods	Scalability	Reproducibility	Solvent Concerns	Drug Loading Efficiency (%)	References
Direct Dissolution	Good–excellent	Moderate	Excellent (no/benign solvent)	Variable (Low–mid)	[68,74,97]
Dialysis	Poor	Moderate	Good–excellent (depends on volume/time/membrane)	Low–moderate	[76,104,105]
Emulsification & Evaporation	Good	Moderate	Poor	Good	[98,106]
Thin-Film Hydration	Moderate	Moderate	Moderate–poor	Moderate	[88,107,108]
Microfluidic-Assisted	Excellent	Excellent	Good–excellent	Variable	[99,100]
Supercritical Fluid (SCF)	Good	Excellent	excellent	Good	[35,39,109,110]
Stimuli-Responsive	Moderate	Excellent	Good–excellent	Variable	[1,36,62,63]
PEG-Assisted	Good	Good	Good	Good	[37,41]

Scalability: poor (<50 mL, difficult steps), moderate (10–100 mL routine), good (100 mL–L + controls), excellent (L-scale or continuous demonstrated). Reproducibility: poor (>25% coefficient of variation (CV) for size/DL), moderate (15–25%), good (10–15%), excellent (≤10% CV; PDI drift ≤0.03). Solvent concerns: poor (Class 2/halogenated, hard removal), good (Class 2 with robust removal/PAT), excellent (no/benign solvent). Drug-loading efficiency: typical DL% band achievable for PEG-polyester/taxane-like small molecules (see Table 2 for quantitative exemplars). DL% = loaded drug/(loaded drug + polymer) × 100. Variable: sensitive to polymer MW/architecture, feed ratio, solids, and mixing/flow history.

**Table 4:** Comparative analysis of emerging micelle fabrication methods, focusing on scalability, solvent requirements, precision control, and regulatory alignment. These next-generation strategies address the limitations of conventional techniques and facilitate GMP-compliant production.

Methods	Key Characteristics	Advantages	Limitations	References
Microfluidic-Assisted	Rapid mixing of an organic phase containing the polymer and drug with an aqueous phase	Narrow size distribution, improved batch-to-batch reproducibility, enhanced cytotoxicity	Difficult scale-up, complex optimization, low throughput, solvent compatibility issues	[34,38,99,100,111]
Supercritical Fluid (SCF)	Processing of polymers and drugs using supercritical fluids	Avoidance of toxic solvents, solvent-free or solvent-minimized conditions, formation of dry micellar powders	Requirement for high-pressure equipment, narrow processing window, need for specialized formulations	[35,39,109,110]
Stimuli-Responsive	Self-assembly is triggered or modulated by environmental stimuli (e.g., pH, redox, enzymes)	Site-specific release, reduced systemic toxicity, enhanced therapeutic index	Synthetic complexity, batch variability, undefined pathways, trigger inconsistency	[1,36,40,62,63,64,65]
PEG-Assisted	Co-dissolution of amphiphilic block copolymers and drugs in low-molecular-weight PEGs	Simple protocol, high encapsulation efficiency, solvent-free process, scalability, regulatory alignment	Requires mild heating, PEG–drug solubility mismatch, hydration condition sensitivity	[37,41]

**Table 5:** Micelle-based strategies across different administration routes and their associated therapeutic benefits. This table summarizes representative micellar systems tailored for specific administration routes including intravenous, oral, transdermal, and pulmonary delivery highlighting their applications, formulation strategies, pharmacological effects, and relevant studies. These route-specific micellar approaches have demonstrated potential in enhancing drug solubility, targeting efficiency, and overall therapeutic efficacy.

Administration Route	Key Application	Micelle Strategy	Therapeutic Effect	References
Intravenous (IV)	Systemic anticancer therapy	Core-crosslinked micelles, PEG-assisted micelles	Prolonged circulation, enhanced tumor accumulation, and reduced off-target toxicity	[37,42,49,87]
Oral (PO)	Gastrointestinal delivery of poorly water-soluble drugs	pH-sensitive micelles, transporter-targeted systems	Improved stability and drug solubility, enhanced permeability and bioavailability	[43,46,47]
Transdermal	Non-invasive delivery of lipophilic agents	Hyaluronan-based micelles, lecithin-integrated micelles	Sustained skin deposition, wound healing efficacy, potential in dermatological and regenerative medicine	[45,50]
Pulmonary	Local or systemic lung drug delivery	TPGS/DPPC mixed micelles	Effective aerosolization, increased lung deposition, cytotoxic and antimicrobial activity	[51,54]

## Data Availability

No new data were created or analyzed in this study.

## Abbreviations

The following abbreviations are used in this manuscript:

CMC	Critical Micelle Concentration
DDS	Drug Delivery System
DPPC	Dipalmitoylphosphatidylcholine
DMSO	Dimethyl Sulfoxide
DENA	N,N-diethylniacinamide
EPR	Enhanced Permeability and Retention
FRET	Förster Resonance Energy Transfer
GMP	Good Manufacturing Practice
GEM	Germacrone
HPMA	N-(2-hydroxypropyl) methacrylamide
LCST	Lower Critical Solution Temperature
MAL	Maleimide
MMP	Matrix Metalloproteinase
mPEG	Methoxy Polyethylene Glycol
PGG	poly-(L-γ-glutamyl-glutamine)
P(2-VBOPNA)	poly(2-(4-(vinylbezyloxy)-N-picolylnicotinamide))
Phe	Phenylalanine
P(VBODENA)	poly(2-(4-vinylbenzyloxy)-N,N-diethylnicotinamide)
PCL	Poly(ε-caprolactone)
PDCL	Poly(DL-caprolactone)
PDLLA	Poly(D,L-lactide)
PEG	Polyethylene Glycol
PLA	Poly(lactic acid)
PLGA	Poly(lactic-co-glycolic acid)
PMPC	Poly(2-methacryloyloxyethyl phosphorylcholine)
QbD	Quality by Design
ROS	Reactive Oxygen Species
SAS	Supercritical Antisolvent
SCF	Supercritical Fluid
TPGS	D-α-Tocopheryl Polyethylene Glycol Succinate
uPAR	Urokinase Plasminogen Activator Receptor
